# iASPP is over-expressed in human non-small cell lung cancer and regulates the proliferation of lung cancer cells through a p53 associated pathway

**DOI:** 10.1186/1471-2407-10-694

**Published:** 2010-12-30

**Authors:** Jinfeng Chen, Fei Xie, Lijian Zhang, Wen G Jiang

**Affiliations:** 1 Key laboratory of Carcinogenesis and Translational Research (Ministry of Education) Department of Thoracic Surgery Peking University School of Oncology and Beijing Cancer Hospital & Institute Beijing 100142 PR China; 2 Metastasis & Angiogenesis Research Group Cardiff University School of Medicine Heath Park Cardiff CF14 4XN UK

## Abstract

**Background:**

iASPP is a key inhibitor of tumour suppressor p53 and is found to be up-regulated in certain malignant conditions. The present study investigated the expression of iASPP in clinical lung cancer, a leading cancer type in the world, and the biological impact of this molecule on lung cancer cells.

**Methods:**

iASPP protein levels in lung cancer tissues were evaluated using an immunohistochemical method. *In vitro*, iASPP gene expression was suppressed with a lentvirus-mediated shRNA method and the biological impact after knocking down iASSP on lung cancer cell lines was investigated in connection with the p53 expression status.

**Results:**

We showed here that the expression of iASPP was significantly higher in lung cancer tissues compared with the adjacent normal tissues. iASPP shRNA treatment resulted in a down-regulation of iASPP in lung cancer cells. There was a subsequent reduction of cell proliferation of the two lung tumour cell lines A459 and 95D both of which had wild-type p53 expression. In contrast, reduction of iASPP in H1229 cells, a cell with little p53 expression, had no impact on its growth rate.

**Conclusions:**

iASPP regulates the proliferation and motility of lung cancer cells. This effect is intimately associated with the p53 pathway. Together with the pattern of the over-expression in clinical lung cancers, it is concluded that iASPP plays an pivotal role in the progression of lung cancer and is a potential target for lung cancer therapy.

## Background

The tumour suppressor protein p53 is a transcription factor that responds to oncogenic stress such as DNA damage, oncogene activtaion, γ-irradiation and certain chemotherapeutic drugs that may result in apoptosis and cell-cycle arrest [1,2]. In over half of all of human cancers, *p53 *has been shown to be either lost or mutated. In those tumours in which the *p53*gene is intact, the regulation of the p53 pathway may be defect [3,4]. The type of response following p53 activation depends upon a number of factors. Importantly, oncogenic transformation can cause a switch in the cell's response to p53 activation from growth arrest to programmed cell death. As a result, tumour cells are more likely to undergo apoptosis following p53 activation than the corresponding normal cells, making the p53 pathway an excellent target for therapeutic intervention [5-8].

iSAPP, Inhibitory Member of the ASPP (Apoptosis-stimulating protein of p53) family is also known as the Rela-associated inhibitor, RAI and NF-kappa-B-interacting protein-1, NKIP1. It is one of the conserved inhibitors of p53. The discovery of the ASPP family of proteins as specific regulators of p53 identifies a new mechanism by which the apoptotic function of p53 is regulated [9,10]. The name of the family is based on the domain organization of the proteins (ankyrin repeat, SH3, and proline-rich domain containing protein) as well as their functions (apoptosis-stimulating protein of p53) [11]. There are three family members in humans: ASPP1, ASPP2, and iASPP. ASPP1 and ASPP2 enhance the apoptotic function of p53, whereas iASPP inhibits p53-dependent apoptosis [9-13]. Regulatory function of p53 by iASPP is conserved from worm to human [14]. The expression levels of ASPP proteins in human malignancies have been sparsely reported. While ASPP1 and ASPP2 are down-regulated in a large percentage of tumours, iASPP has been found to be significantly higher in patients with acute leukaemia when compared with healthy donors or patients with leukaemia but with complete remission. iASPP has also been found to be over-expressed in breast carcinomas [14-19]. There has been no reports on the expression of the ASPP family and their possible functions in lung cancer.

In the present study, we first investigated the protein expression of iASPP in human lung cancer tissues and further evaluated the impact of knocking down iASPP, by way of lentivirus shRNA to iASPP, on the function of a panel of lung cancer cell lines which exhibited different p53 expression pattern.

## Methods

### Cell lines, reagents and antibodies

Human lung cancer cell lines A549, 95D and H1229 were purchased from the American Type Culture Collection (ATCC, Manassas, VA, USA) and cultured either in in F-12K medium (A549 cells) or RPMI-1640 medium (95D cells and H1229 cells) containing 10% fetal bovine serum, at 37°C with 5% v/v CO_2_. MTT assay reagents were purchased from DingGuo Biotech (Beijing, China). 5-Bromo-2'-deoxyuridine (BrdU) assay reagents were purchased from Chemicon International (Temecula, CA, USA). Anti-iASPP mAb used for Western blot assay was purchased from Abcam (Boston, MA, USA). Anti-iASPP rAb using for Immunohistochemical assay was purchased from Rockland Immunochemicals, Inc., (Gilbertsville, PA, USA). Anti-GAPDH monoclonal was purchased from Santa Cruz Biotechnology (Santa Cruz, CA, USA).

### Lentivirus-mediated shRNA delivery

Sequences of iASPP shRNA were inserted into the pGCL-GFP lentivirus RNAi expression system. The shRNA containing vectors were transfected together into 293T cells with pHelper1.0 and the lentiviral helper plasmid pHelper2.0 to generate the respective lentiviruses. Viral stocks were collected from the transduced 293T cells and were used to infect A549 cells, 95D cells and H1229 cells. The sequence of iASPP nonsense shRNA was: AATGTACTGCGCGTGGAGA; the sequence of iASPP shRNA was AACACATGGATCTGAAGCAGA. The mRNA and protein levels were measured 72 hrs after cells being infected.

### Quantitative RT-PCR analysis of iASPP expression

Total RNA was extracted and reverse transcribed into cDNA using M-MLV-RTase (Promega, Madison, WI, USA). The resulting cDNA was used for PCR using the SYBR-Green Master PCR Mix (Applied Biosystem, Carlsbad, CA, USA) in triplicates. Primers for qRT-PCR were as follows: iASPP forward primer: GGCGGTGAAGGAGATGAAC; iASPP reverse primer: TGATGAGGAAATCCACGATAGAGA; p53 forward primer: CCTCCTCAGCATCTTATCC; p53 reverse primer: ACAAACACGCACCTCAAA; p21 forward primer: GGGACAGCAGAGGAAGACC; p21 reverse primer: GACTAAGGCAGAAGATGTAGAGC; PUMA forward primer: GACGACCTCAACGCACAG; PUMA reverse primer: CACCTAATTGGGCTCCATCTC. PCR and data collection were performed on the TP800 qPCR System (Takara, Japan). All quantitations were normalized to an endogenous β-actin control. β-actin forward primer: GGCGGCACCACCATGTACCCT; β-actin reverse primer: AGGGGCCGGACTCGTCATACT. The relative quantitation value for each target gene compared to the calibrator for that target is expressed as 2^-(Ct-Cc) ^(Ct and Cc are the mean threshold cycle differences after normalizing to β-actin).

### Western blot

Protein samples prepared from the cells were subjected to SDS-PAGE, transferred to PVDF membranes (Millipore, Kankakee, IL, USA) and detected with appropriate primary antibodies followed by horseradish peroxidase-conjugated goat, anti-mouse or rabbit IgG. The protein signals were detected using SuperSignal West Dura Extended Duration Substrate (Pierce, Rockford, IL, USA).

### MTT assay

All the cells, including those transfected, were grown in exponential phase and detached by trypsin/EDTA treatment. Viable cells (2,000 cells/ml) were plated into 96-well tissue culture plates (100 μl complete medium/well) and cultured at 37°C in 5% CO_2_. At different time points, MTT reagent was added (10 μl per well) and incubated at 37°C for 4 hr. The reaction was stopped with addition of 100 μl DMSO and the optical density was determined at OD570 nm on a multi-well plate reader. Data from three independent experiments were analyzed by student *t *test and p < 0.05 was considered statistically significant.

### BrdU assay

Cells were seeded into 96-well plates (1,500 cells/well) and cultured at 37°C in 5% CO_2_. At different time points, BrdU reagent was added (20 μl/well) and incubated at 37°C for 4 hr. Cells were then fixed in a fixation solution for 30 min. After washing three times with a washing buffer, anti-BrdU antibody was added (50 μl/well) and incubated at 37°C for 1 hr. Following washing, an enzyme conjugated secondary antibody was added (50 μl/well) and incubated at 37°C for a further 30 min. Colour was then developed by incubation with 50 μl TMB substrate for 30 min in dark and the optical density was determined at OD490 nm on a multi-well plate reader. Data from three independent experiments were analyzed by student *t *test and p < 0.05 was considered statistically significant.

### Colony formation assay

Cells were seeded into six-well plates (200 cells/well) (in three duplicate wells) and cultured at 37°C in 5% CO_2_. After two weeks, the cells were fixed with paraformaldehyde for 30 min and then stained with GIEMSA for 10 min. ddH2O was used to wash the cells three times to obtain a clean background. The number of colonies and the cell number in each colony were counted and statistically analyzed.

### Immunohistochemical Staining

Tissues sections (5-μm thick) were dewaxed, followed by quenching the endogenous peroxidase with 3% H_2_O_2 _in methanol for 30 min. Prior to staining, non-specific binding was blocked by incubation with 10% BSA in PBS at 37°C for 1 hr. Tissue sections were incubated with pre-immune IgG or specific antibodies in PBS containing 1% BSA at 4°C overnight, followed by incubation with a horseradish peroxidase-conjugated anti-mouse or rabbit antibody. Colour was then developed by incubation with an ImmunoPure Metal Enhanced Diaminobenzidine (DAB) Substrate kit (Pierce). After each incubation, tissue sections were washed three times in PBS for 10 min. Tissue sections were finally counterstained with hematoxylin. For determination of iASPP immunoreactivity, cytosolic staining of yellowish or brownish granules was graded as follows: 0 for background staining, 1 for faint staining, 2 for moderate staining and 3 for strong staining. In addition, positive staining areas in entire tissue section were graded as follows: 0 for <5%, 1 for 5-25%, 2 for 26-50%, 3 for 51-75%, and 4 for 76%-100%. When combining these two parameters, 0-2 and ≥3 were considered negative and positive staining, respectively.

Statistical analysis was carried using SPSS (version 16). Fisher's Exact test was used for analyzing the immunohistochemical data and Student t test for other quantitative data.

## Results

### Over-expression of the iASSP protein in human lung cancer tissues

**Figure 1:**
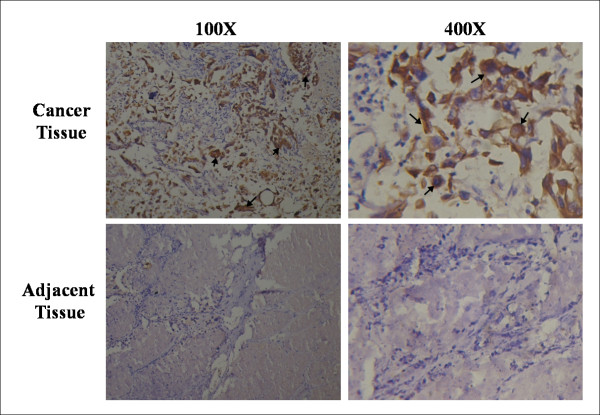
**Over-expression of iASPP in human lung cancer**. Expression of iASPP in cancer tissue (top panels) and adjacent tissue (low panels). Results are representative of more than ten immunohistochemical staining experiments. Positively stained tumour cells are indicated by arrows.

To determine the expression pattern of iASPP protein in human lung cancer, immunohistochemical analysis was performed on 49 pairs of tumour and normal tissues from patients pathologically verified for having lung carcinoma. Immunoreactivity for iASSP antigens was seen in 40.82% (20/49) of lung cancer tissues and 4.35% (2/46) of adjacent non-cancerous tissues (Figure 1), p < 0.001 by Fisher's Exact test. The number of samples that were assigned each score is shown in Table 1. Others previously reported that SNPs in iASPP are related to the response of chemotherapy and radiotherapy in NSCLC (non-small cell lung cancer) patients [20]. Thus, the increase in iASPP expression may play an important role in the pathogenesis of human lung cancer.

**Table 1:** The expression pattern of iASPP in lung cancer samples revealed in immunohistochemistry analysis.

	Cancer tissue		Adjacent tissue	
	
Staining grade*	Case	Percentage	Case	Percentage
<5%	7	14.29%	30	65.22%
5%-25%	10	20.41%	10	21.74%
26%-50%	12	24.49%	4	8.70%
51%-75%	11	22.45%	2	4.35%
75%-100%	9	18.37%	0	0.00%

* Samples were defined as "iASPP positive" when the proportion of tumor cells positive for iASPP (staining rate) was more than 50%.

### Reduction of iASPP mRNA and protein expression by shRNA in vitro

**Figure 2:**
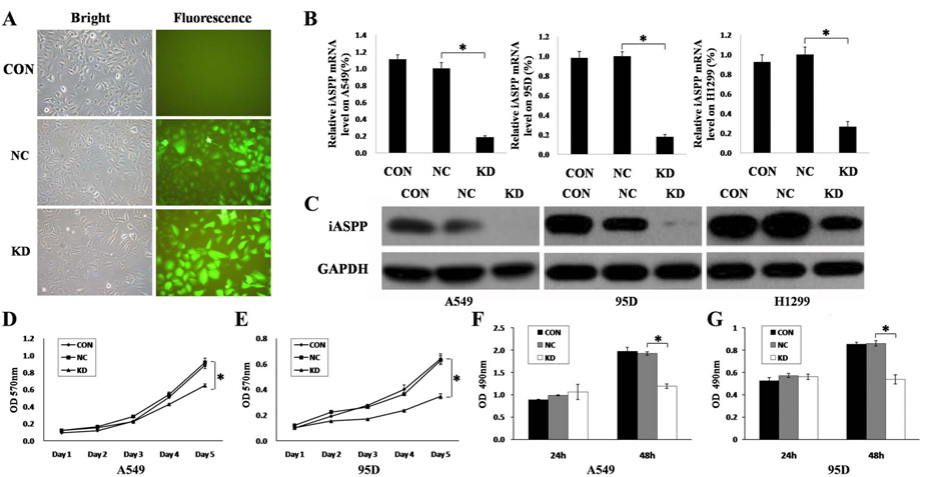
**Down-regulation of iASPP inhibits proliferation of lung cancer cells**. (A) Pictures for Lentivirus tranfection of blank control (CON), nonsense shRNA (NC) and iASPP shRNA (KD). (B) The iASPP mRNA level was downregulated by iASPP shRNA in A549, 95D and H1299 cells. (C) The iASPP protein level was downregulated by iASPP shRNA in A549, 95D and H1299 cells. (D-E) Proliferation of A549 cells (D) and 95D cells (E) were inhibited when treated with iASPP shRNA by MTT assay. (F-G) Proliferation of A549 cells (F) and 95D cells (G) were inhibited when treated with iASPP shRNA by BrdU assay. CON: blank control; NC, nonsense shRNA; KD: iASPP shRNA. Results represent the mean ± S.D. of three independent experiments.

To further investigate the biological role of iASPP in lung cancer cells, we knocked down iASPP transcript in human lung cancer cell lines A549 cells, 95D cells and H1229 cells. This was carried out by employing shRNA technology. shRNA to iASPP was constructed into PGCL-GFP vector using lentivirus transfection system, as shown in Figure 2A. The lentivirus transfection system successfully down-regulated iASPP expression at both mRNA level and protein level in A549 cells, 95D cells and H1229 cells, in comparison with blank controls or nonsense shRNA controls (Figure 2B, C).

### iASPP down-regulation inhibited the proliferation of A549 cells and 95D cells

Using the iASPP knockdown cells, we first tested the change of cell growth, using the MTT assay. The results showed a significant decrease of cell growth and indicated that cell proliferation was inhibited after transfection with iASPP shRNA both in A549 cells and 95D cells (Figure 2D, E). To further conform this result, we carried out an alternative proliferation assay, the BrdU assay. Consistent with the observations made with the MTT assay, both A549 cells and 95D cells showed lower BrdU positive staining after the cells were treated with iASPP shRNA than the blank controls or cells treated with nonsense shRNA for 48 hours (Figure 2F, G). Our results thus suggested that shRNA mediated down-regulation of iASPP inhibited proliferation of lung cancer cells. It is interesting to observe that BrdU assay appeared to be a more sensitive method than the MTT assay. A significant difference was seen as early as 48 hours with the BrdU assay (Figure 2D), whereas with the MTT assay (Figure 2F), the difference was seen after 4 days.

### iASPP down-regulation inhibited the colony formation of A549 cells

**Figure 3:**
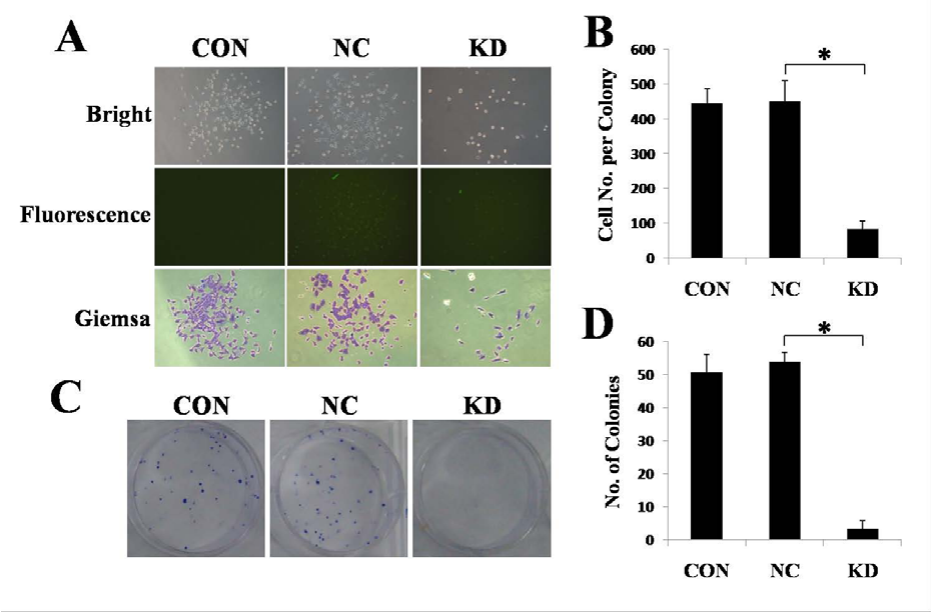
**Down-regulation of iASPP inhibits colony formation of lung cancer cells**. (A-B) iASPP shRNA (KD) reduced the number of cells in a signal clonal colony compared with the blank control (CON) or nonsense shRNA (KD). (C-D) iASPP shRNA (KD) also reduced the number of cells compared with the blank control (CON) or nonsense shRNA (KD). Results represent the mean ± S.D. of three independent experiments.

Transfection of A549 cells with iASPP shRNA resulted in a significant decrease in cell numbers in each colony, when compared with A549 cells treated with blank control or nonsense shRNA (Figure 3A, B). The number of colonies with more than 50 cells was also decreased in iASPP shRNA transfected cells (Figure 3C, D). 95D cells were unable to form colonies and were unable to be assessed for this cellular function (data not shown).

### iASPP regulated lung cancer cell proliferation, a connection with the p53 pathway

**Figure 4:**
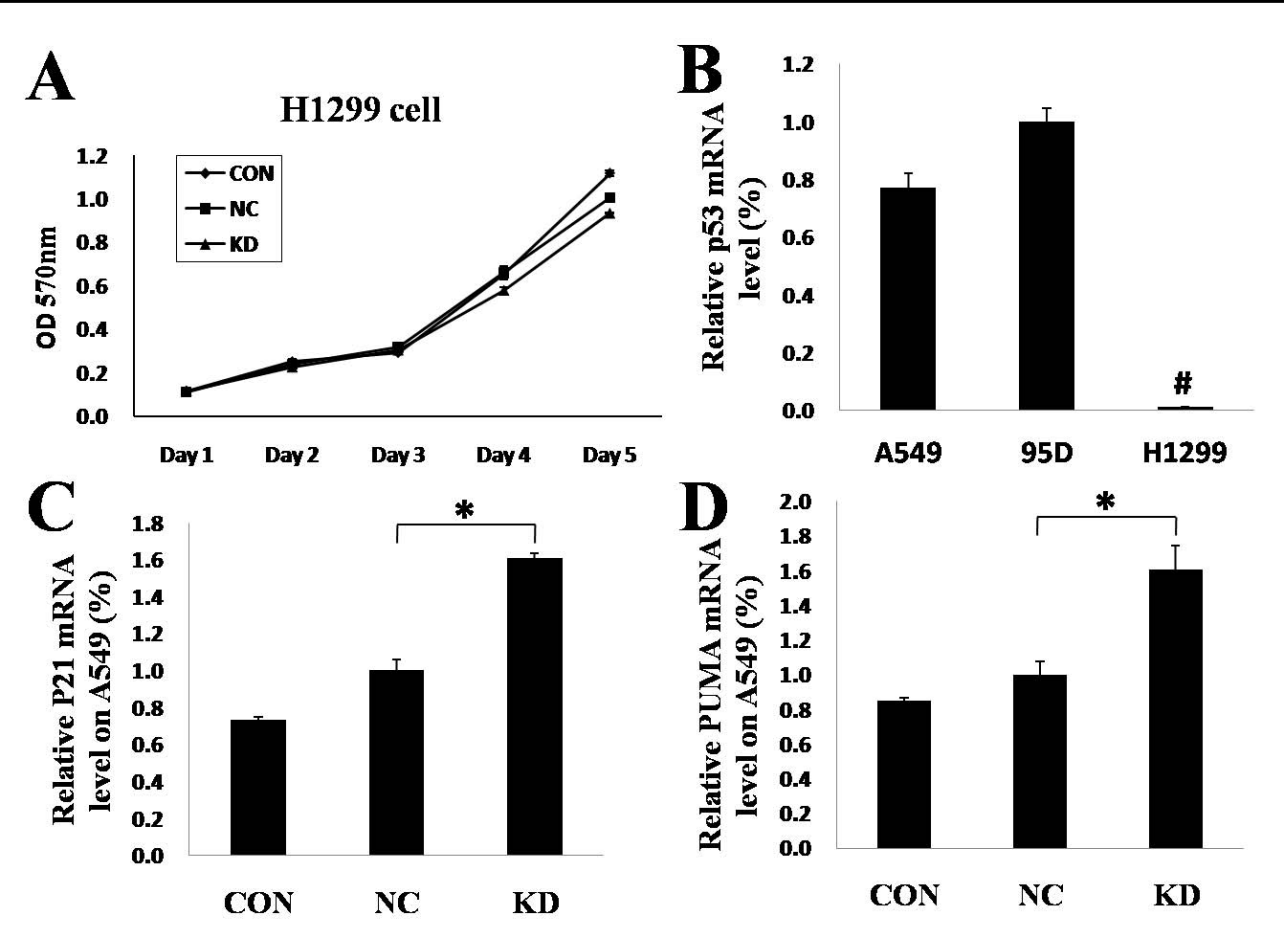
**Down-regulation of iASPP inhibits proliferation of lung cancer cells through p53**. Down-regulation of iASPP did not affect the proliferation of H1229 cells. (B) A549 cells and 95D cells had high p53 mRNA level, whereas H1299 cells had very low p53 mRNA levels. (C-D) The mRNA level of p21 and PUMA, which was downstream of the p53 pathway, was up-regulated when treated with iASPP shRNA in A549 cells. CON: blank control; NC, nonsense shRNA; KD: iASPP shRNA. Results represent the mean ± S.D. of three independent experiments.

Although iASPP down-regulation inhibited the proliferation of A549 cells and 95D cells, there was no changes in the proliferation of H1229 cells when treated with iASPP shRNA, as evident from both MTT and BrdU assays (Figure 4A, and data not shown). Since iASPP has been reported as a regulator of p53, we thus suspected that the difference between A549/95D cells and H1229 cells in their response to iASPP shRNA may be due to different p53 expression. Consistent with our hypothesis, it was found that the H1229 cells expressed little iASSP mRNA, whereas A549 cells and 95D cells showed high levels of the wild-type iASPP mRNA (Figure 4B). As a downstream effector of p53 activation, p21 and PUMA [21,22] were also found to be significantly increased in A549 cells after treatment with iASPP shRNA, when compared with blank control or nonsense shRNA (Figure 4C, D). This provides further support that iASPP regulates lung cancer cell proliferation in a manner that is associated with the p53 pathway.

## Discussion

To the best of our knowledge, the present study is the first report of up-regulation of iASPP in human lung cancers. Our study has shown that there was an over-expression of the iASPP protein in lung tumour tissues in comparison with normal tissues and that knocking down iASPP resulted in an inhibition of cell proliferation. Given the prevalent of lung cancer and the death rate associated with the tumour type [23], our finding has important bearing.

In accordance with our results, others have reported that SNPs in iASPP are associated with response to chemotherapy or combined chemotherapy and radiotherapy in NSCLC (non-small cell lung cancer) patients [20]. Together, it suggests that iASPP plays important roles in lung cancer. In the present study, we also demonstrated that iASPP down-regulation inhibited the proliferation and colony formation of lung cancer cell lines A549 and 95D *in vitro*. A few recent reports have indicated that iASPP is over-expressed in breast cancers and certain types of leukaemia, and that down-regulation of iASPP could inhibit the proliferation of these cancer cells [14-19]. Together with our results in lung cancer, it is plausible to suggest that iASPP acts as a common factor of regulating the proliferation in difference cancer cells. Over-expression of iASPP may be involved in both the establishment and the progression of tumours, whereas down-regulation of iASPP may inhibit tumour development. In this regard, drugs specifically directed against iASPP could be beneficial in the treatment of cancers, including lung cancer [24].

p53 is perhaps one of the best known tumour suppressor genes and plays a critical role in regulating cell proliferation through induction of growth arrest or apoptosis. iASPP is the most phylogenetically conserved inhibitor of p53 identified thus far. Our results suggesting that iASPP down-regulation in H1229 cells did not affect the proliferation and colony formation is very interesting. H1229 cells have little p53 expression as shown in the literature and in the present study. This thus confirms that the effect of iASPP is largely dependent upon p53 in order to regulate the apoptotic pathway, as seen with other cancer cell types. Besides p53, the p53-related protein p73 also has the similar functions. Previously, iASPP has been shown to form a protein complex with p73 to regulate cell death [8,24]. Whether iASPP forms the same complex with p73 in lung cancer cells deserve further exploration.

The ASPP family consists of three members, ASPP1, ASPP2 and iASPP. All three proteins share sequence similarity at their C-terminus which contains their signature sequences of ankyrin repeats, SH3 domain and Proline-rich region. The C-terminus is the preferred binding site for p53 [25]. In contrast to iASPP, ASPP1 and ASPP2 activate p53 to stimulate specifically the expression of pro-apoptotic target genes. This implies that ASPP1 and ASPP2 can compete with iASPP for binding to p53, thereby inhibiting the ability of iASPP and stimulating the apoptotic function of p53. However, the detailed mechanism requires further exploration.

## Conclusions

We report that that iASPP is over-expressed in human non-small cell lung cancer tissues when compared with normal tissues. shRNA mediated down-regulation of iASPP results in inhibition of proliferation and colony formation of two lung tumour cell lines A459 and 95D which have high wild-type p53 expression [26,27] and has no impact on the H1229 cells which has little p53 expression *in vitro*. This further suggests that iASPP is a target for lung cancer therapy associated with p53 pathway.

## Competing interests

The authors declare that they have no competing interests.

## Authors' contributions

JC and FX contributed equally to the study design, experimental work, data analysis and preparation of the manuscript. LZ and WGJ contributed to the study design, data analysis and manuscript preparation. All the authors read and approved the manuscript.

## Pre-publication history

The pre-publication history for this paper can be accessed here:

http://www.biomedcentral.com/1471-2407/10/694/prepub
